# Angiogenesis in cancer of unknown primary: clinicopathological study of CD34, VEGF and TSP-1

**DOI:** 10.1186/1471-2407-5-25

**Published:** 2005-03-03

**Authors:** Vasilis Karavasilis, Vasiliki Malamou-Mitsi, Evangelos Briasoulis, Elena Tsanou, Evangelia Kitsou, Haralambos Kalofonos, George Fountzilas, Theodore Fotsis, Nicholas Pavlidis

**Affiliations:** 1 Medical Oncology Department, Ioannina University Hospital, Ioannina, Greece; 2 Sarcoma Unit, Royal Marsden Hospital, London, UK; 3 Department of Pathology, Ioannina University Hospital, Ioannina, Greece; 4 Medical Oncology Department, Patras University Hospital, Rion, Greece; 5 Medical Oncology Department, School of Medicine, Aristotle's University of Thessaloniki, Greece; 6 Laboratory of Biochemistry, School of Medicine, University of Ioannina, Ioannina, Greece; 7 Biomedical Research Institute-Foundation for Research and technology (BRI-FORTH), Ioannina, Greece

## Abstract

**Background:**

Cancer of unknown primary remains a mallignancy of elusive biology and grim prognosis that lacks effective therapeutic options. We investigated angiogenesis in cancer of unknown primary to expand our knowledge on the biology of these tumors and identify potential therapeutic targets.

**Methods:**

Paraffin embedded archival material from 81 patients diagnosed with CUP was used. Tumor histology was adenocarcinoma (77%), undifferentiated carcinoma (18%) and squamous cell carcinoma (5%). The tissue expression of CD34, VEGF and TSP-1 was assessed immunohistochemically by use of specific monoclonal antibodies and was analyzed against clinicopathological data.

**Results:**

VEGF expression was detected in all cases and was strong in 83%. Stromal expression of TSP-1 was seen in 80% of cases and was strong in 20%. The expression of both proteins was not associated with any clinical or pathological parameters. Tumor MVD was higher in tumors classified as unfavorable compared to more favorable and was positively associated with VEGF and negatively with TSP-1.

**Conclusion:**

Angiogenesis is very active and expression of VEGF is almost universal in cancers of unknown primary. These findings support the clinical investigation of VEGF targeted therapy in this clinical setting.

## Background

Cancer of unknown primary (CUP) is a unique clinical entity that accounts for an approximately 3% of human cancers[1]. Patients with CUP present with metastases for which the site of origin cannot be identified at initial workup. Early dissemination, unpredictability of metastatic pattern and aggressiveness constitute fundamental characteristics of these tumors. Although the clinical characteristics of CUP have been established, little is known about the underlying biology of these tumors [2,3].

Angiogenesis, the formation of new vessels, is essential for tumor growth and the development of metastases. It evolves though a complex multifactor process that involves interaction of pro-angiogenic and anti-angiogenic signals from tumor, endothelial and stromal cells. The angiogenic activity is reflected in the development of novel microvessels in tumor tissue that is quantified by the intratumoral microvessel density (MVD). Among several molecules implicated, Vascular Endothelial Growth Factor (VEGF) and Thrombospondin-1 (TSP-1) appear to be most relevant. Much evidence indicates that VEGF is a key activator of angiogenesis[4,5] and TSP-1 a primary endogenous inhibitor of angiogenesis[6] Up to now, no useful prognostic factors have been established other than the classic pathologic and laboratory ones and immunohistochemical detection of various factors did not add prognostic value in CUP.[7,8] Moreover, investigation of the expression of crucial angiogenesis factors that can be therapeutically targeted is today of great interest for the oncologists who deal with CUP clinical research.[9]

We were prompted to investigate angiogenesis in unknown primary cancer in an attempt to enrich our understanding of the biology of these tumors. We studied by immunohistochemistry the tissue expression of VEGF and TSP-1 in CUP and correlated with MVD and clinicopathological parameters. In a recently published study vascular endothelial growth factor, and CD34, factors were not found to be of prognostic value in adenocarcinoma of unknown primary.[8]

## Methods

A total of 81 patients diagnosed with CUP and treated in three University Medical Oncology Settings (Ioannina, Patras and AHEPA, Thessaloniki, Greece) between January 1997 and December 2002 were selected on the basis of availability of archival tumor tissues and accessibility to medical notes. Pathology diagnosis was reviewed by two pathologists blinded to written pathology report and representative paraffin blocks were selected for immunohistochemistry.

### Subgroup definition

Eligible cases categorized into unfavorable and more favorable subgroups (tables 1 and 2). Patients with poorly differentiated carcinoma with midline distribution, papillary adenocarcinoma of peritoneal cavity and adenocarcinoma involving only axillary lymph nodes in women, squamous cell carcinoma involving cervical lymph nodes and poorly differentiated neuroendocrine carcinomas were assigned to favorable CUP subsets. Patients with adenocarcinoma metastatic to the liver, multiple visceral involvement and extensive metastatic bone disease were considered as unfavorable.

**Figure 1:**
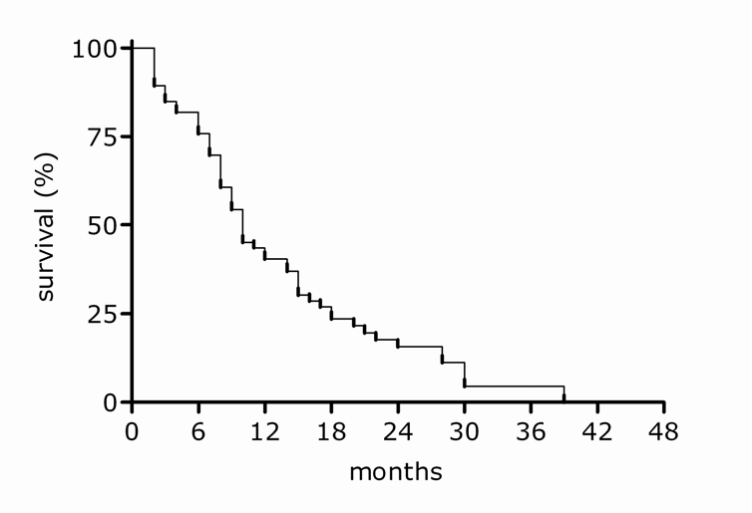
Survival curve of 81 CUP patients analyzed

Systemic chemotherapy was given in 64 patients (78%); four patients with cerebral metastases received whole brain irradiation. Chemotherapy was consisted of a platinum based combination. Objective response to chemotherapy was observed in 34 patients (53%) while one patient with brain metastases responded to radiotherapy. Median survival for all patients was 10.5 months (Figure 1). Patients belonging to favorable subsets had a significantly higher response rate to treatment (Fisher's t-test, *p *= 0.04) and a longer survival, 11.5 vs 8.5 months (*p *= 0.01).

### Immunohistochemistry

Immunostaining was performed on formalin-fixed, paraffin-embedded tissue sections by the labeled streptavidin avidin biotin (LSAB) method. In brief, tissue sections were deparaffinised in xylene and dehydrated. They were immersed in citrate buffer (0,1 m, pH 0,6) and subjected to microwave twice for 15 min. Subsequently, all sections were treated for 30 min with 0,3% hydrogen peroxide in methanol to quench endogenous peroxidase activity. Mouse monoclonal antibodies directed against human CD34 antigen (M 7165, Dako) in dilution 1/50 β) VEGF Ab-3 (isoform 121, clone Jh121, Neomarkers) in dilution 1/50 and c) thrombospondin (Mob 315, DBS) in dilution1/50 were used. Positive control slides were included in all cases. All dilutions were made in TBS-1% BSA solution and were followed by overnight incubation.

The assessment of immunostaining was made by two experienced pathologists using light microscope. Tumor specimens too small to provide sufficient sections for all the immunoassaying procedures were disregarded from the study.

### Immunostaining evaluation

Staining of endothelial cells for CD34 was used to evaluate the MVD. Any CD34 positive endothelial cell clusters clearly separated from each other were considered as single countable microvessels. A lumen was not required to identify a vessel. Larger vessels with muscular walls were excluded from counting. In each sample three areas of most prominent vascular density (hot spots) were identified at ×40 power field and microvessel counting was done under ×400 magnification. Counting was performed by two independent observers blinded to clinical information. The median count was used to make distinction between low and high MVD.

Immunoreactivity for VEGF was observed in stromal and epithelial cells. Only staining of tumor cells was considered for analysis. To evaluate the expression of VEGF protein, we devised a combined score that corresponds to the sum of staining intensity (0 = negative, 1 = weak, 2 = intermediate, 3 = strong staining) and percentile quadrants of positive cells (0 = 0%, 1 = 1–25%, 2 = 26–50%, 3 = >50%). The maximum score was 6. Score 2 was regarded to represent weak expression, score 3 intermediate and score 4–6 strong expression.

Staining for TSP-1 was only considered in regard to extracellular matrix. The expression of TSP-1 was characterized according to the extent and the intensity of staining classified as negative, +1: mild, focal, +2: intermediate, multifocal, +3: strong, diffuse reactivity.

### Statistics

Staining results were analyzed against clinical subgroup, histological differentiation, response to treatment and survival. The association between MVD and clinical subgroup, histological differentiation and response to treatment was assessed by an unpaired t test. A Fisher's exact test was used to determine associations between VEGF and TSP-1 and the clinical subgroup, histological differentiation and response to treatment. Spearman non parametric correlation test was used for associations between MVD, VEGF and TSP-1.

Survival was calculated by Kaplan-Meier method and comparison of survival curves was performed by the log-rank test. For statistical significance a two-tailed p value was considered. The Graphpad Instat version 3.05 (Graphpad Software, Inc, San Diego, CA) and Prism version 4 (Graphpad Software, Inc, San Diego, CA) software programs were used for statistical analysis and graphing.

## Results

Demographics of studied cases are depicted in Tables 1 and 2. Overall survival of patients included in this study is shown in Figure 1. (Median survival 10 months).

### Immunohistochemistry

#### Microvessel density

**Figure 2:**
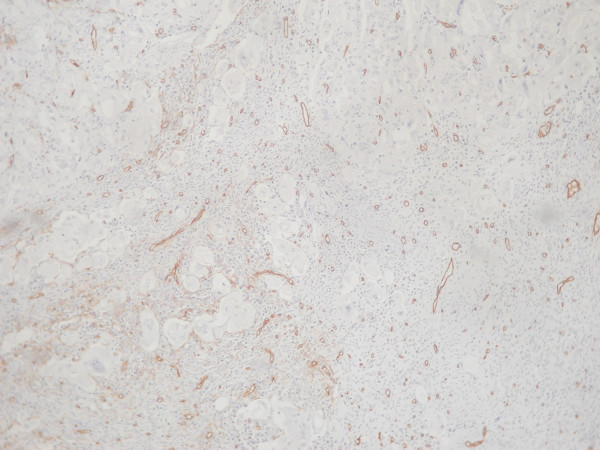
High-density of neoplastic vessels highlighted by stained anti-CD34 antibody in a case of a poorly differentiated adenocarcinoma of unknown primary (Original magnification 200, counterstained with hematoxylin).

Widespread staining for CD34 was seen in all tumor specimens. Within the most prominent vascular areas of the tumors the recorded mean MVD was 59 microvessels/mm^2 ^(range, 16 to 300 microvessels/mm2) (Table 3, figure 2). A positive association was observed between VEGF expression and MVD (Spearman r = 0.36, p = 0.0016) and negative with TSP-1.

#### VEGF expression

**Figure 3:**
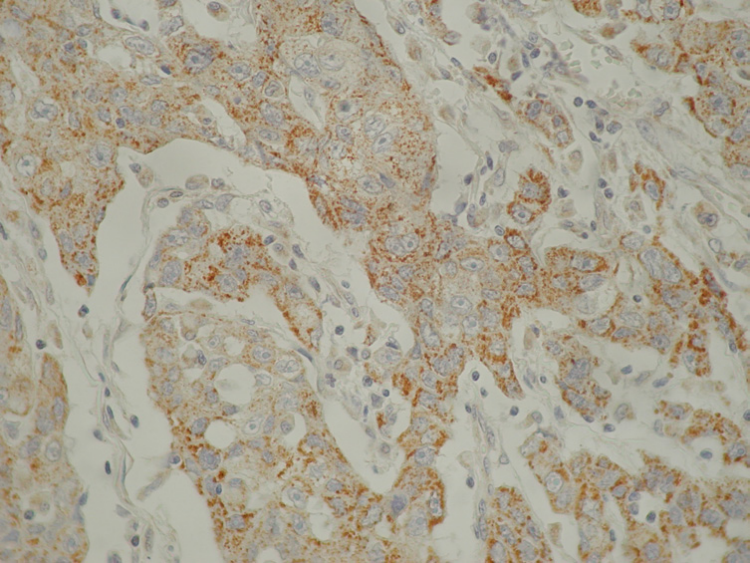
Strong cytoplasmic immunohistochemical staining of tumor cells for VEGF in a CUP case. It is shown the characteristic granular cytoplasmic staining pattern and the occasionally weak positive reaction of intervening stromal and endothelial cells (original magnification ×200, counterstained with hematoxylin)

Positive staining of tumor cells for VEGF, both membranic and cytoplasmic was observed in all cases. This was strong in 83% of the cases and moderate in 17%. VEGF staining was heterogeneous within tumors comprising of areas of intense and also weak staining and demonstrated a characteristic granular cytoplasmic staining pattern. (Figure 3) A weak and focal positive reaction of intervening stromal cells (fibroblasts or/and macrophage) and endothelial cells was also seen in some cases. This staining was excluded from VEGF analysis. (Table 3)

#### TSP-1 expression

**Figure 4:**
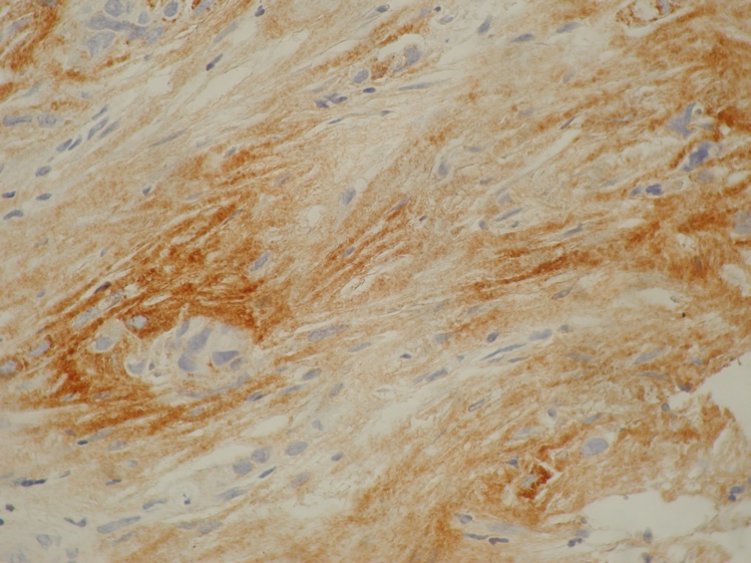
Diffuse strong immunoreactivity of extracellular matrix for TSP-1 adjacent to neoplastic cell population in a CUP case. (Original magnification ×400, counterstained with hematoxylin)

Stromal TSP-1 staining was detected in 80% of the cases, while in 20% it was absent (figure 4). Strong (score = 3)and intermediate (score = 2) TSP-1 staining was observed in 50% of cases and weak (score = 1) in 30% (Table 3). A negative association was observed between TSP-1 expression and MVD, (Spearman r = -0.3426, p = 0.003) while there was no association between TSP-1 and VEGF expression.

#### Association between immunostaining and clinicopathological variables

MVD was found statistically higher in unfavorable CUP cases compared to more favorable ones (70 vs 46 microvessels/mm2, t test, p = 0.034) (table 4). This was the only correlation detected between angiogenesis related tissue markers studied and clinicopathological variables. No association was detected between VEGF and TSP-1 and tumor differentiation, response to treatment, clinical subgroups and survival (Table 4).

## Discussion

The investigation of the biological profile of CUP and the understanding of molecular pathways underlining these tumors has been limited. We have worked on these issues and found several oncoproteins overexpressed, but failed to establish any clinically relevant correlations[10,11]. We now investigated neo-angiogenesis by assessing MVD and the tissue expression of two representative molecules involved in angiogenesis; the major stimulator of angiogenesis VEGF (A), and the intrinsic angiogenic inhibitor TSP-1. Overall, we demonstrated that a high angiogenetic activity occurs in CUP tumors, which is higher in unfavorable when compared with more favorable subsets.

VEGF is known to play a key role and MVD is considered to reflect the final result of the tumor angiogenesis cascade. In the present study, all cases were found to be VEGF-positive and in the majority VEGF was overexpressed. Tumor VEGF and MVD were strongly correlated that is in line with findings in solid tumors[12,13]. We failed to demonstrate any significant correlations of angiogenic activity with regard to clinical outcome, but this was obviously due to universal expression of both CD34 and VEGF in our cases.

We also demonstrated that in our series TSP-1 was overexpressed in 50% and was absent or weak in approximately half of the cases. TSP-1 correlated inversely with microvessel counts. The role of TSP-1 in epithelial tumor growth and metastases remains controversial. In vitro studies suggest that TSP-1 may promote tumor cell adhesion and invasion by up-regulating urokinase plasminogen activator and its receptor[14] but  in clinical studies overexpression has been associated with a lower MVD score and a better clinical outcome in several carcinomas[15]. Moreover, other studies suggest that TSP-1 inhibits tumor progression and may serve as an indicator of less aggressive potential and of favorable prognosis in solid tumors.[16] We consider that low TSP-1 in our material reflects a suppression of anti-angiogenic mechanism of TSP-1 that possibly contributes to the aggressiveness of these tumors.

CUP patients have in general a brief life expectancy with a median survival approximately of 3–9 months.[17,18] It must be emphasized that CUP diagnosis applies to a heterogeneous group of patients who are usually grouped together in biological and therapeutic studies to obtain statistically meaningful results. However several patients fare better and enjoy longer survival and within this more favorable prognostic subgroup, unique subsets, such us young patients with midline tumors and women with peritoneal carcinomatosis or isolated axillary adenocarcinoma, have a distinct clinical biology compared to others also classified as unknown primary cancer. [19-23]. In our study MVD score were found low in the group of more favorable tumors compared to unfavorable, but neither MVD nor VEGF or TSP-1 were associated with known prognostic factors.[24] Similarly, Hillen et al, in a small study, evaluated MVD as a prognostic factor for patients with liver metastases of unknown primary and found that MVD score correlated with marginally shorter survival.[25]

## Conclusion

In conclusion, we found that angiogenesis is very active and VEGF expression is universal in cancer of unknown primary, which supports the clinical investigation of VEGF targeted therapy in this clinical setting.[9] To identify additional druggable molecular targets in cancer of unknown primary we need to advance our knowledge on the biology of these tumors and validate novel molecular therapeutics.

## Competing interests

The author(s) declare that they have no competing interests.

## Authors' contributions

NP conceived, coordinated and designed the study, interpreted the data and drafted the manuscript; VK, EB, designed and carried out the study, performed the statistical analysis, interpreted the data and drafted the manuscript; VMM carried out the pathological and immunohistochemical study, interpreted data and drafted the manuscript; ET, EK carried out the pathological and immunohistochemical study, interpreted data and drafted the manuscript; HK, GF, TF participated in designing the study, acquisition and interpretation of data and revising critically the manuscript. All of the authors have read and approved the final manuscript.

## Pre-publication history

The pre-publication history for this paper can be accessed here:



## Figures and Tables

**Table 1:** Demographics.

**Characteristic**	**No**	**(%)**
Patients	81	
Sex		
Male	39	48
Female	42	52
Age (Years)		
Median	66	
Range	37–84	
PS		
Median	1	
Range	0–3	
Histology		
Adenocarcinoma	62	77
*Well- and moderately differentiated*	*27*	*43*
*Poorly differentiated*	*35*	*57*
Undifferentiated carcinoma	15	18
*With neuroendocrine features*	*5*	*33*
*Other undifferentiated neoplasms*	*11*	*67*
Squamous cell carcinoma	4	5
Metastatic sites at presentation		
Liver and/or multiple visceral involvement	17	21
Lymph nodes	23	29
*Mediastinal-retroperitoneal*	*17*	*74*
*Axillary*	*2*	*9*
*Cervical*	*4*	*17*
Peritoneal cavity	19	23
*Peritoneal adenocarcinomatosis in females*	*14*	*74*
*Malignant ascites and other site*	*5*	*26*
Lung	5	6
Bones	5	6
Brain	4	5
Neuroendocrine tumors	4	5
Multiple	4	5

**Table 2:** CUP subsets and outcome.

**Subgroups**	**(#)**	**(%)**
Favorable	50	62
Unfavorable	31	38
**Treatment**	**(#)**	**(%)**
Chemotherapy	64	78
Radiotherapy	4	6
**Median survival**	**(months)**	
All cases	10.5	
favourable cases	11.5	
unfavorable cases	8.5	

**Table 3:** Assessment of tissue expression of VEGF, TSP-1 and CD34.

	VEGF	TSP-1	MVD (CD34)
Immunostaining score			

Median	5	2	59 microvessels/mm2
(range)	2–6	0–3	16–300

Expression intensity (% of cases)			

Negative	0	20	
Weak	0	30	
Intermediate	17	30	
Strong	83	20	

**Table 4:** Immunohistochemical expression of CD34 (MVD). VEGF, and TSP-1 and correlations.

*Variable*	*MVD (counts)*		*VEGF (%)*			*TSP-1 (%)*		
	Median CD 34	*P value*	Low (<4)	High (≥4)	*P value*	Low (<2)	High (≥2)	*P value*

**Age**		*ns*			*ns*			*ns*
< 65	62		20	80		35	27	
> 65	58		15	85		22	16	
**Tumor differentiation**		*ns*			*ns*			*ns*
Moderate and well	55		3	28		12	20	
Poorly and undifferentiated	59		14	55		32	36	
**Clinical subgroups**		*0.034*			*ns*			*ns*
More favorable	46		6	46		28	23	
Unfavorable	70		11	37		22	27	
**Treatment outcome**		*ns*			*ns*			*ns*
Objective response	58		11	39		22	28	
No response	59		4	46		25	25	
